# Energy‐Efficient Actuation for Wearable Exoskeletons: A Virtual Prototype

**DOI:** 10.1155/abb/7248941

**Published:** 2025-11-21

**Authors:** Asim Ghaffar, Abdur Rehman, Muhammad Tanveer Riaz, M. M. Sayed Al Mahmud

**Affiliations:** ^1^ Department of Mechanical, Mechatronics and Manufacturing Engineering, University of Engineering and Technology Lahore, Faisalabad Campus, Faisalabad, Pakistan, uet.edu.pk; ^2^ Department of Basic Sciences and Humanities, University of Engineering and Technology Lahore, Faisalabad Campus, Faisalabad, Pakistan, uet.edu.pk; ^3^ Department of Electrical and Electronic Engineering, US-Bangla Assets Ltd., Dhaka, 1212, Bangladesh

## Abstract

This study investigates the power consumption of an assistive wearable exoskeleton actuation system using a virtual experimental framework. Different actuation system variants, including rigid, series elastic, and parallel elastic actuation in both single and dual configurations, were analyzed and compared with a mathematical model. The results demonstrated a strong correlation between the virtual and mathematical models, with only minor variations in power consumption across certain transmission system combinations. The study further found that combining harmonic drives with a belt and pulley mechanism resulted in reduced energy usage. Among the configurations analyzed, the dual variable parallel elastic actuation (VPEA) system, featuring harmonic drives at the hip and knee joints and ball screws at the ankle, proved to be the most energy‐efficient setup. These findings validate the accuracy of the mathematical model and offer valuable guidelines for optimizing exoskeleton actuation systems to enhance their efficiency and performance.

## 1. Introduction

In recent years, the advancement of assistive robotic exoskeletons has seen notable progress, driven by their promising applications in areas such as rehabilitation [1, 2], mobility assistance [3, 4], and performance augmentation [5]. These wearable robotic systems are designed to enhance human movement by providing controlled actuation at key joints, thereby reducing physical effort and improving mobility for individuals with disabilities or motor impairments [6]. However, designing an effective exoskeleton requires addressing critical challenges related to weight, power efficiency, and actuation performance [7, 8]. A well‐balanced system must be lightweight for user comfort, powerful enough to provide adequate assistance, and energy‐efficient to extend operational time without frequent battery recharging [9].

To optimize these factors, researchers have explored various actuation mechanisms, including rigid actuation, elastic actuation, and hybrid configurations [10–12]. Each approach presents trade‐offs in terms of power consumption, torque generation, and compliance, necessitating rigorous analysis and validation before practical implementation [13, 14]. While mathematical models provide theoretical insights into actuation dynamics, real‐world implementation often reveals unforeseen complexities that must be addressed through simulation‐based validation and virtual prototyping [15].

This study introduces the design and assessment of a virtual prototype for a wearable assistive exoskeleton robot, intended to validate and improve mathematical models related to actuation dynamics. By leveraging a digital twin approach, the virtual prototype enables the testing of multiple actuator configurations under realistic conditions, eliminating the need for immediate physical fabrication [16, 17]. The study considers various actuation models, including a dual‐motor configuration, which integrates both rigid and elastic actuation principles to optimize efficiency and assistive performance.

To ensure precise motion control, a PID‐regulated DC motor is implemented, along with pulse width modulation (PWM) techniques to enhance speed modulation efficiency [18]. Several transmission mechanisms, including harmonic drives [19], ball screws [20], and belt‐pulley systems [21], are incorporated to analyze their impact on power efficiency and mechanical response [22]. Furthermore, torque sensors [23], rotational motion sensors, and power consumption monitors are embedded in the simulation framework to enable a comprehensive performance evaluation.

A key aspect of this study is the comparison of virtual prototype simulation results with theoretical predictions from existing mathematical models [24–26]. By analyzing key performance metrics such as torque output, power consumption, and mechanical compliance, this work seeks to establish a strong correlation between simulation outcomes and analytical expectations [27]. Additionally, different elastic actuation configurations, including series and parallel elastic elements [28–32], are explored to assess their potential in reducing energy consumption, while maintaining effective assistive capabilities.

The findings of this research offer valuable guidance on selecting the most effective actuation setup for a high‐performance, energy‐efficient robotic exoskeleton. Results indicate that the variable parallel elastic actuation (VPEA), when paired with harmonic drives and belt‐pulley systems at the hip and knee joints, along with ball‐screw mechanisms at the ankle, achieves an optimal balance between energy efficiency and assistive capability. Although this configuration involves added complexity due to extra actuators and transmission elements, the resulting efficiency improvements make it a worthwhile trade‐off for real‐world use.

By leveraging advanced simulation techniques, this paper contributes to the broader field of wearable robotics by providing a structured framework for virtual prototyping, actuator selection, and energy optimization. The insights gained pave the way for the next‐generation exoskeletons, which can be lighter, more efficient, and better suited for rehabilitation and mobility assistance applications.

## 2. Methodology

### 2.1. Components and Virtual Prototyping of an Assistive Robotic Exoskeleton

The exoskeleton model was initially conceptualized and designed in SolidWorks before being integrated into a multibody dynamic simulation environment for comprehensive analysis.

### 2.2. Virtual Prototype Development

**Figure 1:**
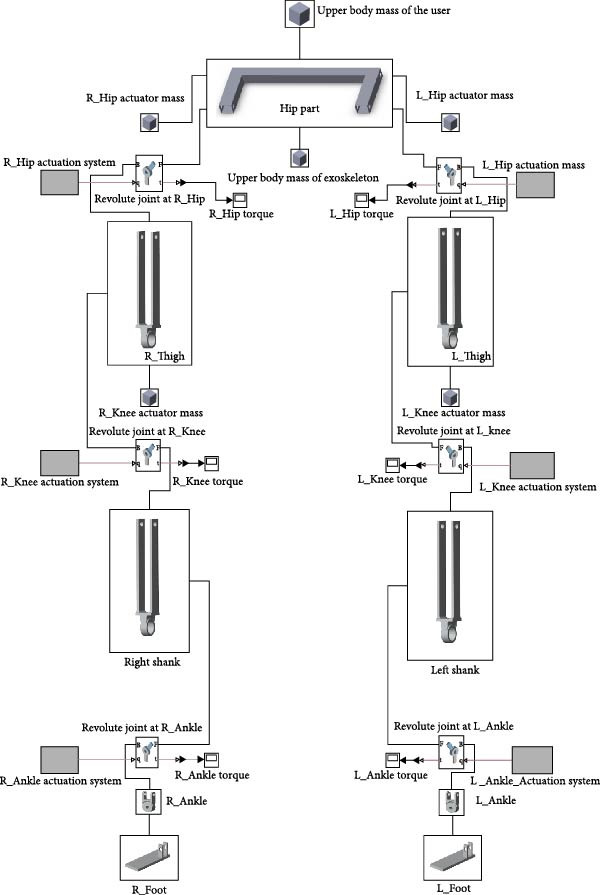
Model of an assistive robotic exoskeleton.

After importing the model into the virtual prototyping and analysis framework, the actuation system was integrated to simulate the exoskeleton’s movement. A global reference frame was assigned to the left foot, and components were interconnected as per the original SolidWorks assembly. Each part was represented as a block, which encapsulated its mass, dimensions, joint locations, and inertia properties, computed automatically for accurate simulation. The resulting model of an exoskeleton is shown in Figure 1.

### 2.3. Actuation System and Integration

To enable movement, six actuators were incorporated at the three primary lower‐limb joints of both legs. Each joint was assigned a mass block to represent the weight of its corresponding actuator. In the hip assembly, two additional mass blocks were incorporated to represent the user’s upper‐body weight and the mass of the upper portion of the exoskeleton. The remaining lower‐body mass was distributed proportionally across the relevant limb segments.

The actuator components were modeled and linked to the exoskeleton’s joints via revolute joints. Each actuator system consisted of a controller, motor driver, DC motor, transmission mechanism, sensor, and in some cases, an elastic element. A PID controller was implemented to regulate motor speed, ensuring minimal deviation from the desired values.

To optimize performance, transmission mechanisms were incorporated to reduce motor size and enhance torque output. Sensors were utilized to measure the actual joint speed, enabling a real‐time comparison with the target values. Each actuation system was fine‐tuned using prerecorded parameters for efficient operation.

### 2.4. Actuation System Architecture

**Figure 2:**
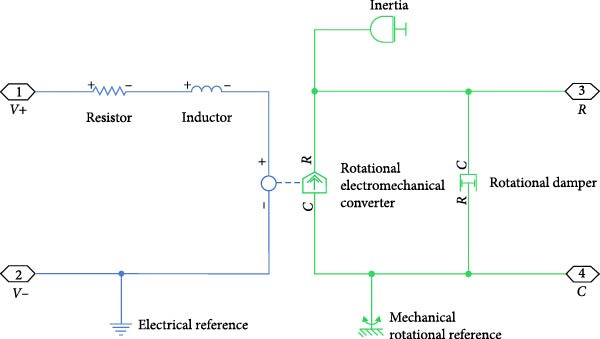
Schematic representation of a DC motor.

**Figure 3:**
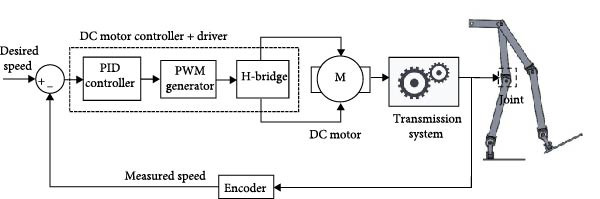
Block diagram of joint actuation system using PWM.

The assistive robotic exoskeleton utilizes DC motors as the primary actuators across all joints, modeled in MATLAB Simscape using physical blocks to replicate electrical and mechanical behavior (Figure 2). These motors are driven using a PWM‐based control scheme generated by a microcontroller, where motor speed is regulated through duty cycle modulation. Direction control is achieved using an H‐bridge. A PID controller closes the feedback loop, adjusting motor input based on measured angular speed to track the desired profile. The control structure is illustrated in Figure 3.

To ensure robust performance across all primary joints, the PID controllers were independently tuned for the hip, knee, and ankle joints under each actuation mechanism (rigid, elastic, and dual motor). The tuning process involved initial auto‐tuning in MATLAB Simulink, followed by manual adjustments based on trajectory tracking performance and system response.

Table 1 below summarises the final tuned PID gains for each joint and actuation mechanism:

**Table 1:** Joint‐wise PID Gain values for different actuation mechanisms.

Actuation type	Joint	*K*_*P*_	*K*_*I*_	*K*_*D*_
Rigid	Hip	82	59	47
	Knee	75	52	44
	Ankle	68	50	39

Elastic	Hip	70	55	45
	Knee	66	50	40
	Ankle	64	48	35

Dual motor	Hip	60	48	38
	Knee	58	46	36
	Ankle	62	45	34

*Note:* For the dual motor and elastic configurations, the PID gains were tuned to account for compliance or load sharing, which naturally damps system dynamics, allowing slightly lower gains while maintaining tracking performance.

**Figure 4:**
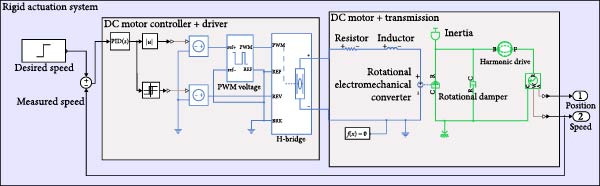
Rigid actuation system with harmonic drive as the transmission mechanism.

**Figure 5:**
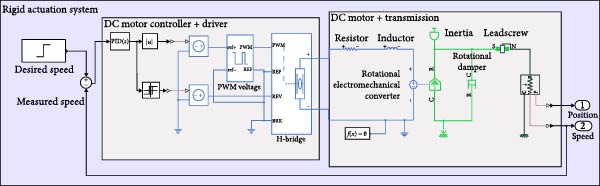
Rigid actuation system using lead screw as the form of the transmission mechanism.

**Figure 6:**
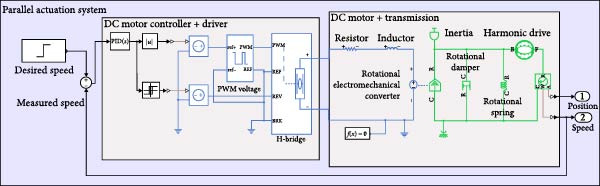
An elastic system using a parallel spring in a virtual prototype of an actuation system.

Furthermore, system‐level models for each actuation configuration were implemented in MATLAB Simscape. Each model includes the motor, driver, sensing elements, transmission mechanism, and joint dynamics. A representative schematic for each actuation mechanism is shown in Figures 4–6, providing a clear view of system architecture and control loop integration.

To increase torque and customize actuation profiles, the motors are coupled with different transmission systems: harmonic drives (Figure 4), belt‐pulley mechanisms and lead screws (Figure 5). Harmonic drives offer compact high‐ratio gearing, modeled using Equation (1) with the gear ratio *N*.

(1) N=nenc−ne,

where *n*_*e*_ denotes the tooth count of the elliptical gear and *n*_*c*_ represents the tooth count of the circular ring gear.

Belt drives provide further reduction, while lead screws convert rotary motion to linear output.

Sensor elements, including voltage, current, speed, and torque sensors, were integrated for control and performance monitoring. Ideal sensor models were used, omitting noise and delay for simplicity since power and control performance were the primary metrics.

To simulate variable compliance, elastic actuation systems were developed using torsional springs arranged either in parallel or in series with the motor (Figure 6). series elastic actuators (SEAs) are commonly used in exoskeleton systems to provide safe and precise force control. They consist of a motor connected to the load through an elastic element, such as a spring, which allows the actuator to store and release energy for smoother and more compliant movements. By measuring the spring’s deflection, the system can accurately control the force applied to the user, enhancing comfort, safety, and adaptability during human–robot interaction. The spring stiffness and initial preload were tuned based on prior optimization results. Additionally, dual‐motor actuation systems were implemented to share the load between two motors, with added elastic elements enabling variable stiffness by adjusting spring parameters before task execution. The motors operated at half the original speed while delivering full torque via a belt‐pulley transmission. This modular actuation framework enables a unified comparison across rigid, elastic, and dual configurations for sit‐to‐stand (STS) and walking tasks.

### 2.5. Optimal Control‐Based Comparison of Actuation Mechanisms

To strengthen the analysis of energy‐efficient actuation and address real‐time deployability, we compared traditional PID control with optimal control techniques for each of the three actuation mechanisms: rigid, elastic, and dual motor systems. The comparison was carried out on the hip joint, as it represents the largest torque demands in lower‐limb motion.

#### 2.5.1. Linear System Modeling

The state‐space representation for each actuation type was derived based on its physical modeling in Simscape. The general model form is as follows:

x˙=Ax+Bu,

(2) y=Cx,

where *x* includes joint angle and velocity, and *u* is the control input (motor voltage or torque).

For elastic actuators, the spring deflection (*θ* − *θ*_*m*_) was modeled as an additional state, introducing coupling dynamics. Dual actuation was modeled with split torque inputs and additional inertia components.

#### 2.5.2. Controller Design

• PID control was tuned manually.
• Linear quadratic regulator (LQR) control was implemented using MATLAB’s lqr() function, minimizing:

(3) J=∫0∞xTQx+uTRudt.

• Model predictive control (MPC) was implemented with a 10‐step prediction horizon and constraints on motor torque and joint position, reflecting actuator limits.

#### 2.5.3. Performance Metrics

The three control methods (PID, LQR, and MPC) were evaluated using the following metrics for each actuation type: (i) Tracking accuracy, quantified by the root mean square error (RMSE) between the intended and actual trajectories of the joints; (ii) control effort, assessed through the total torque applied by the actuators over the duration of the motion; and (iii) energy consumption, calculated by integrating the electrical power input to the motors over time. These metrics provide a comprehensive basis for evaluating both motion fidelity and efficiency of the control schemes under various actuation conditions.

## 3. Results and Discussion

This section summarizes the results from the designed setup and analyzes the performance of the various actuation system variants discussed earlier. Additionally, the power consumption of the virtual actuation system will be investigated for rigid, elastic, and dual rigid‐elastic actuation systems to assess their efficiency and effectiveness.

### 3.1. Performance Assessment of Actuation System Variants

The output of the actuation system using different DC motor models was evaluated. Two primary variants were considered: one built using basic physical components and another incorporating PWM for speed control. Additionally, a direct approach, where the desired joint speed was applied without an actuation system, was implemented for comparison. The performance of these systems was analyzed during STS (STS) and walking maneuvers, with a focus on STS for illustration.

#### 3.1.1. Hip Joint Performance

The mechanical and electrical power of the DC motor at the hip joint during STS was analyzed. Mechanical power was derived from rotational speed and torque, while electrical power was calculated from voltage and current. The results from the three actuation models are shown in Figure 7.

Figure 7(a) Rotational speed, (b) voltage, (c) torque, (d) current, (e) mechanical power, and (f) electrical power of several variants of the virtual prototype of an actuation system for the hip joint during sit‐to‐stand (STS) maneuver.

**Figure:**
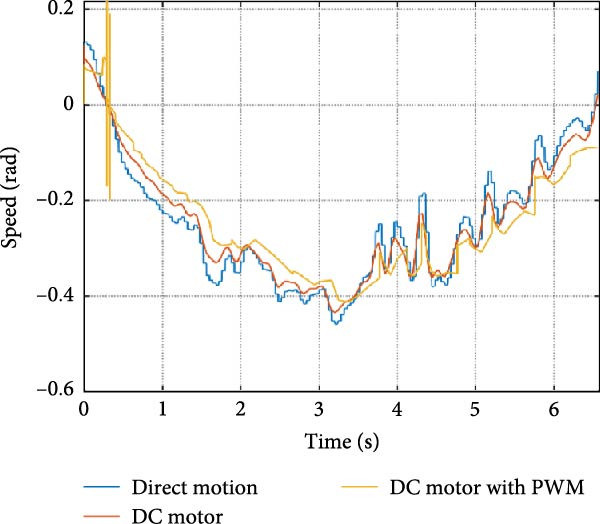
(a)

**Figure:**
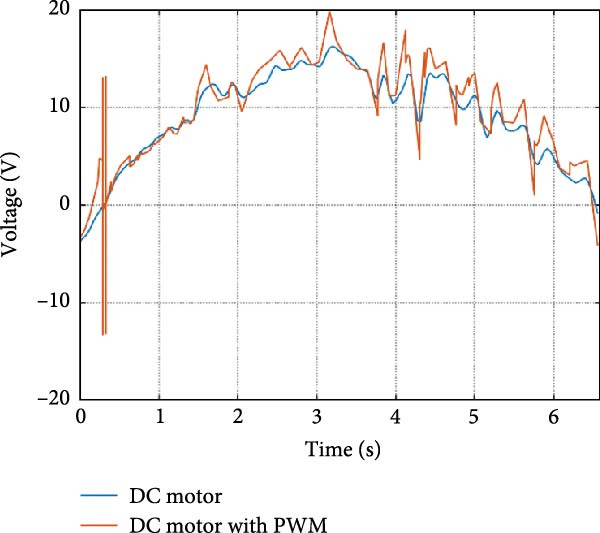
(b)

**Figure:**
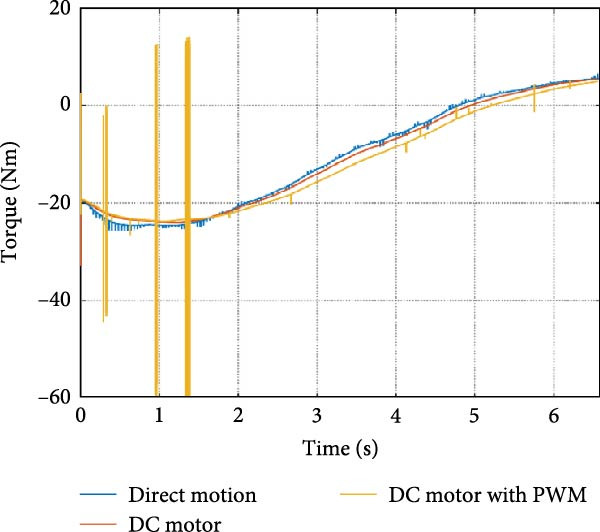
(c)

**Figure:**
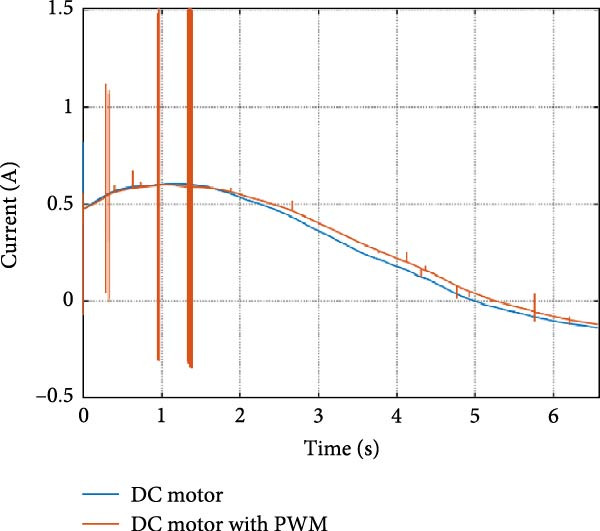
(d)

**Figure:**
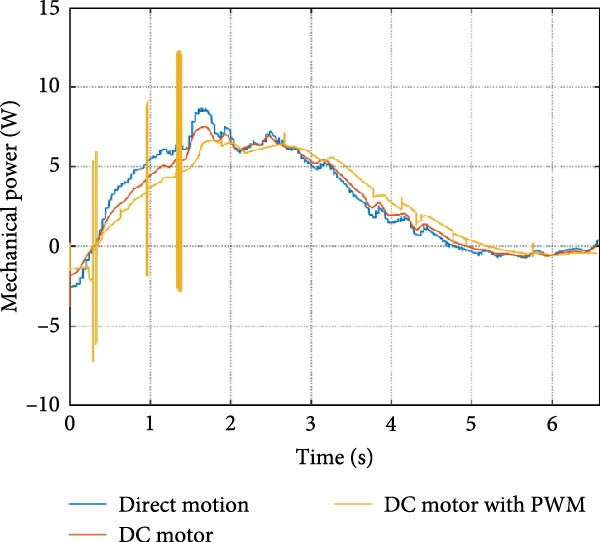
(e)

**Figure:**
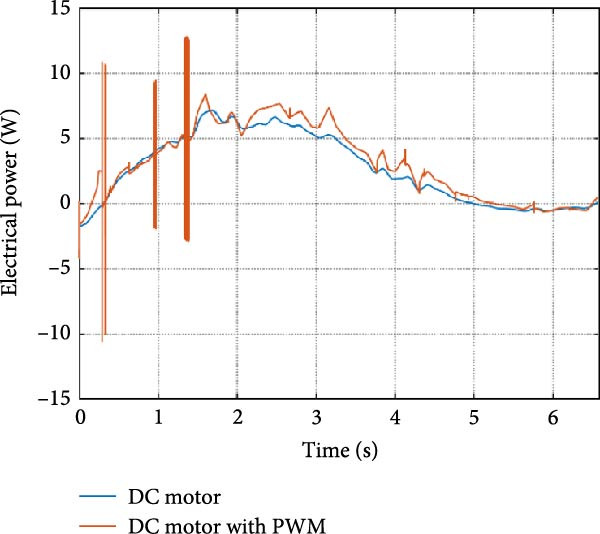
(f)

The direct motion approach produced noisy and oscillatory outputs. The second variant, incorporating a DC motor with a controller, resulted in a smoother torque output due to closed‐loop feedback. The PWM‐based actuation system also demonstrated a smooth output but with random noise fluctuations due to the PWM signal frequency.

Additionally, the simulation time increased with higher model complexity, requiring a trade‐off between model fidelity and computational efficiency. The voltage and current signals followed a similar pattern, exhibiting occasional random spikes in the full actuation model. However, the overall mechanical and electrical power trajectories remained consistent across all approaches.

#### 3.1.2. Knee Joint Performance

Figure 8 shows the actuation system output at the knee during the STS maneuver. All three actuation models displayed trends similar to those observed at the hip joint.

Figure 8(a) Rotational speed, (b) voltage, (c) torque, (d) current, (e) mechanical Power, and (f) electrical power of several variants of the virtual prototype of an actuation system at the knee joint during sit‐to‐stand (STS) Maneuver.

**Figure:**
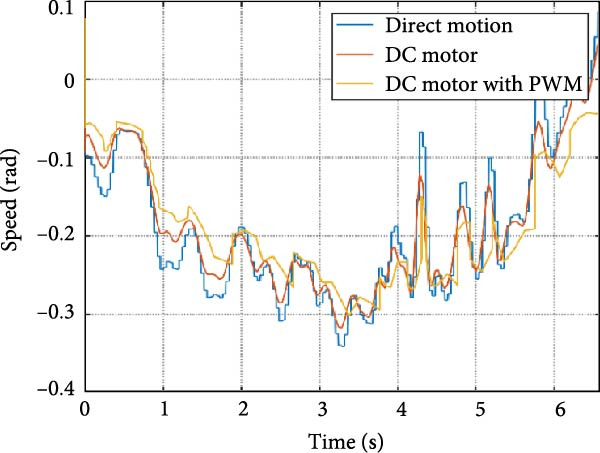
(a)

**Figure:**
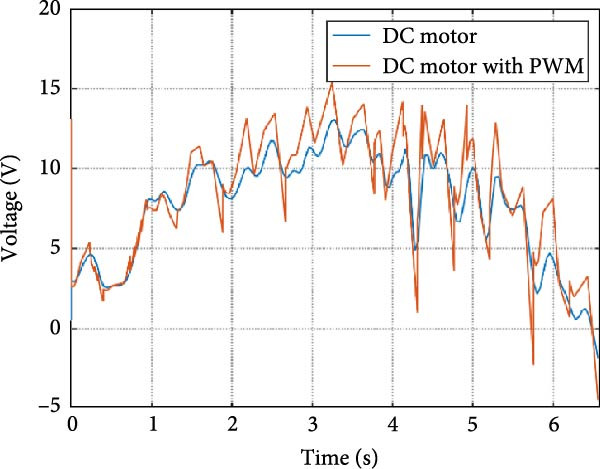
(b)

**Figure:**
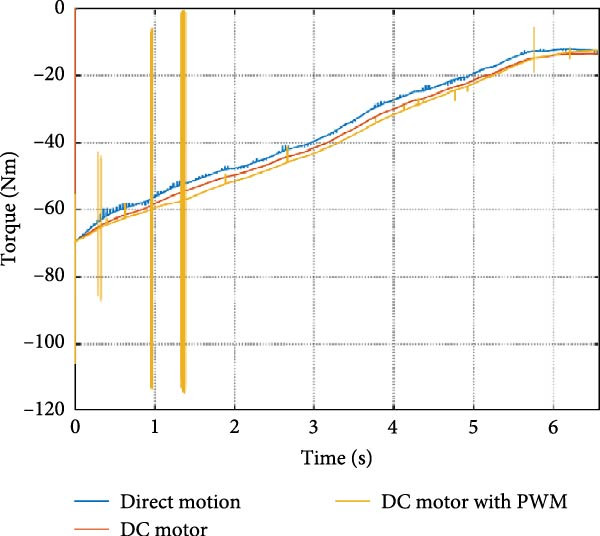
(c)

**Figure:**
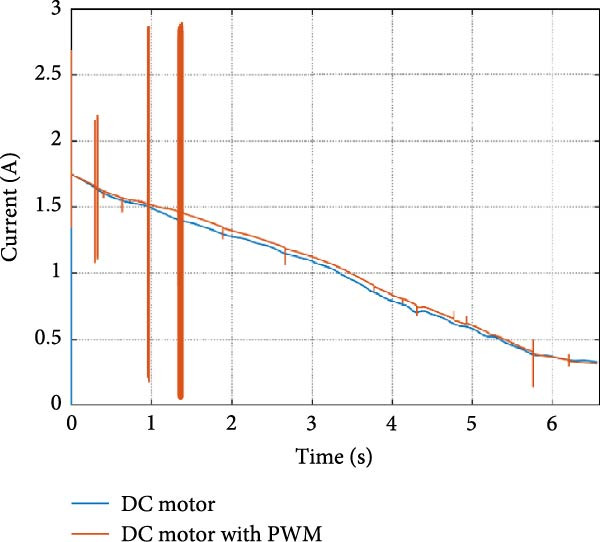
(d)

**Figure:**
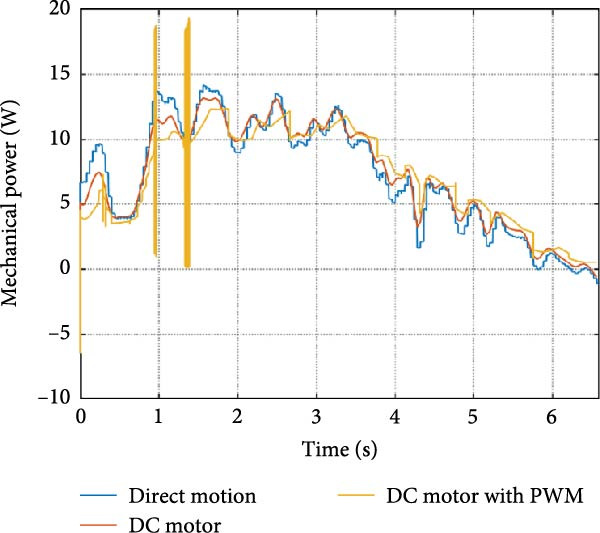
(e)

**Figure:**
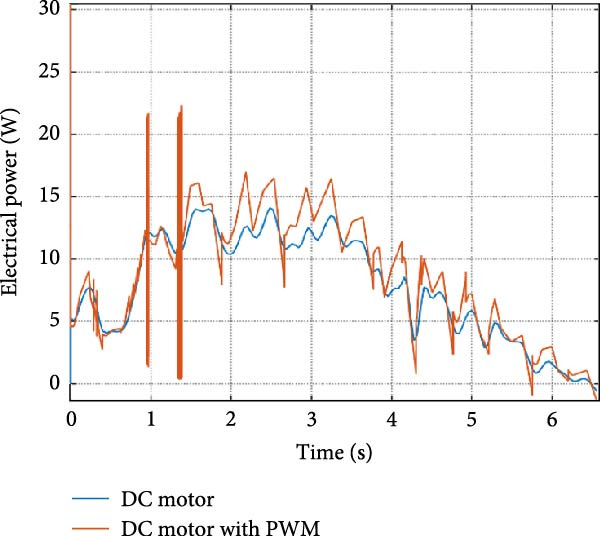
(f)

The direct motion approach resulted in a smooth speed curve, but spikes were observed in the torque trajectory. The second variant, utilizing a DC motor with a controller, produced a smoother response with fewer oscillations across all parameters. Additionally, the simulation time was shorter due to the simpler model structure. The full actuator model displayed smooth speed and voltage curves, but random spikes and oscillations appeared in the torque and current signals. The electrical and mechanical power of the DC motor followed a similar pattern, indicating consistent system behavior across different actuation approaches.

#### 3.1.3. Ankle Joint Performance

Figure 9 illustrates the mechanical and electrical parameters of the actuation system at the ankle joint during the STS maneuver.

Figure 9(a) Rotational speed, (b) voltage, (c) torque, (d) current, (e) mechanical power, and (f) electrical power of several variants of the virtual prototype of an actuation system at the ankle joint during sit‐to‐stand (STS) Maneuver.

**Figure:**
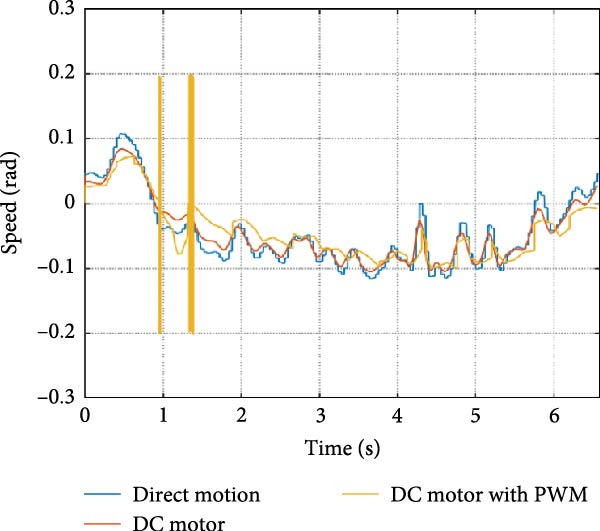
(a)

**Figure:**
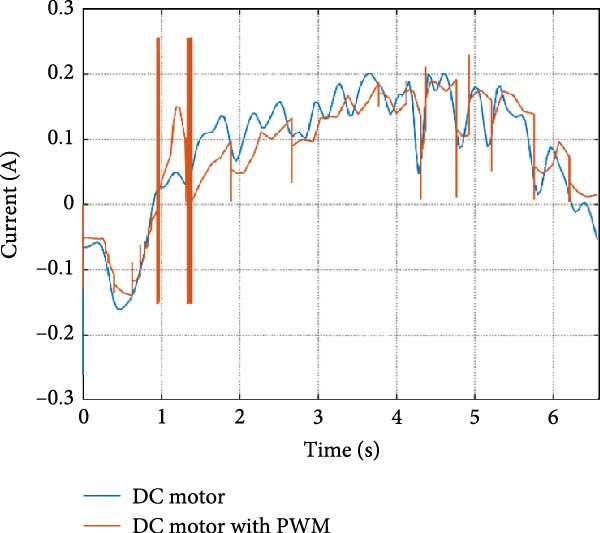
(b)

**Figure:**
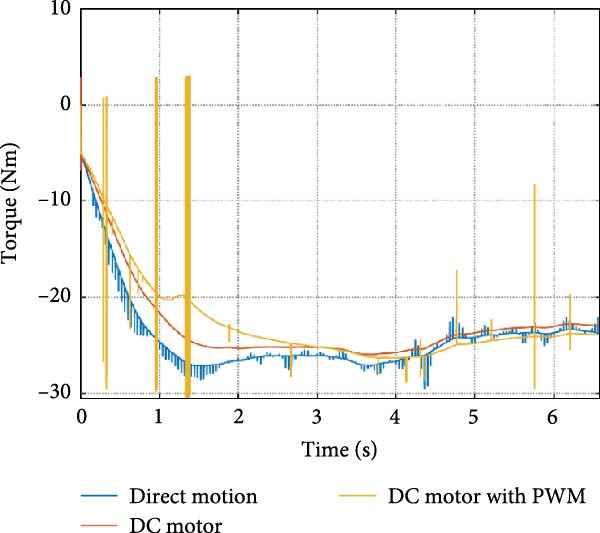
(c)

**Figure:**
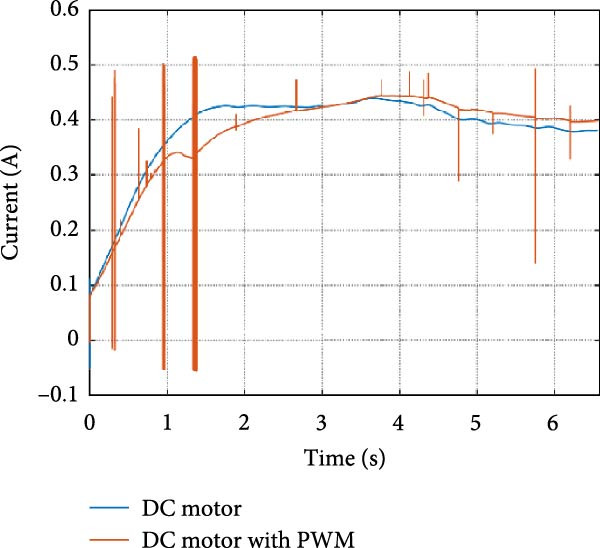
(d)

**Figure:**
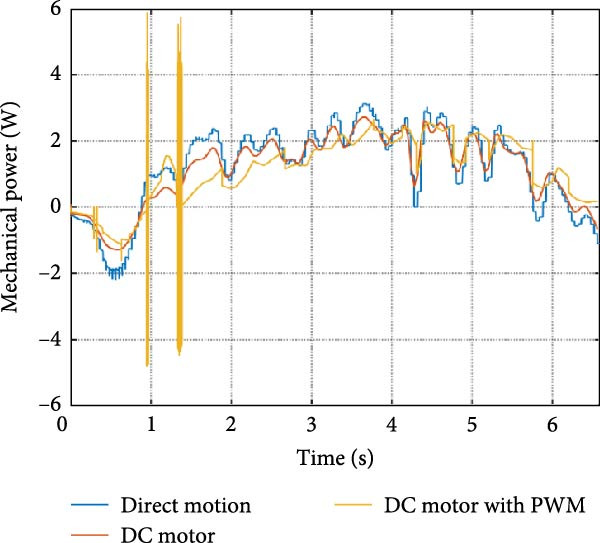
(e)

**Figure:**
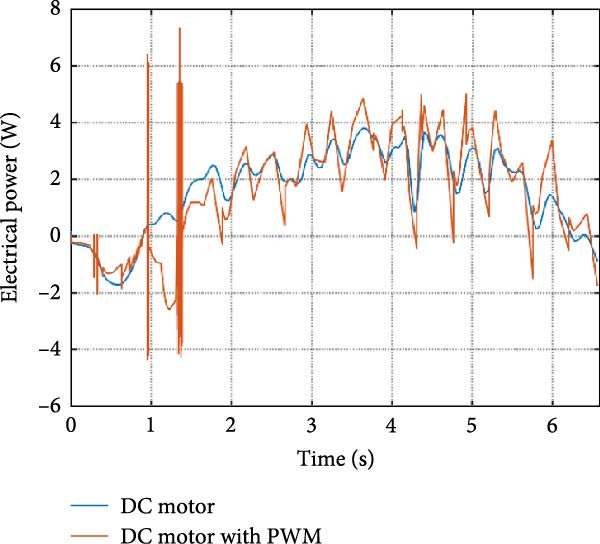
(f)

In the direct motion model, the speed curve was smooth, but spikes were observed in the torque trajectory and mechanical power. Transitioning to the actuator model, the parameters became smoother with fewer spikes. The full actuator model also exhibited smooth graphs with some random spikes, though simulation time increased due to added system complexity.

The trajectory patterns remained consistent across different maneuvers, reinforcing the reliability of the system. The direct motion approach produced idealized speed and position curves, but large spikes appeared in the torque curve. These spikes diminished as the system progressed towards the actuator model, resulting in smoother torque and power curves.

The simulation results in Figure 7e,f show that the mechanical power output starts off lower than the electrical power input, but during later peaks, the mechanical power surpasses the electrical power. This behavior is characteristic of simulation environments where certain losses such as friction, heating or electrical resistance are either idealized or significantly reduced. In the initial phase, the electrical energy is primarily used to overcome inertial effects and accelerate the system. As the motion continues, mechanical power benefits from stored kinetic or elastic energy, particularly in elastic and dual actuation systems, which can temporarily exceed electrical input due to ideal backdriving and energy return in the model. It is important to note that in real‐world systems, this effect would be less pronounced due to energy losses not present in the simulated model.

An inverse relationship between motor current and output torque was observed in Figures 7c,d, 8c,d, and 9c, d across all actuation systems. This behavior can be attributed to the control dynamics and power regulation within the simulated PID‐based actuation framework. In particular, the closed‐loop controller adjusts current input to maintain desired trajectory or speed, even if instantaneous torque requirements are low. Conversely, during phases of increased torque demand, mechanical assistance from stored kinetic or inertial energy may reduce current draw. This leads to a decoupling between torque and current profiles, especially prominent in simulated systems with idealized components and lossless power transmission.

The exoskeleton successfully followed the trajectory in all three cases. However, optimizing actuation system parameters proved to be a complex design challenge, requiring trade‐offs in simulation time, control complexity, and system efficiency. The full actuation model significantly increased simulation time due to the incorporation of detailed physical components and power electronics.

For efficiency, simple controllers were used, avoiding inter‐controller coupling, which could have improved overall system performance. Additionally, the exoskeleton’s trajectory was predefined and did not account for external disturbances such as rough terrain or external forces.

### 3.2. Comparative Analysis of Control Methods for Actuation Systems

Tables 2 and 3 summarize the performance of three actuation mechanisms—rigid, elastic, and dual motor systems under three control strategies: PID, LQR, and MPC, for STS and level ground walking (LGW) tasks. The evaluation metrics include trajectory tracking error (RMSE), total energy consumption, and maximum actuator torque.

**Table 2:** Results for sit‐to‐stand (STS) motion.

Actuation type	Controller	RMSE (rad)	Total energy (J)	Max torque (Nm)
Rigid	PID	0.072	14.3	18.6
	LQR	0.048	12.1	16.4
	MPC	0.035	11.2	15.9

Elastic	PID	0.064	13.0	17.3
	LQR	0.041	11.0	15.2
	MPC	0.030	10.2	14.8

Dual	PID	0.058	12.5	16.0
	LQR	0.038	10.6	14.3
	MPC	0.028	9.5	13.7

**Table 3:** Results for level ground walking (LGW).

Actuation type	Controller	RMSE (rad)	Total energy (J)	Max torque (Nm)
Rigid	PID	0.065	12.8	16.7
	LQR	0.044	10.6	14.5
	MPC	0.032	9.7	13.8

Elastic	PID	0.057	11.4	15.2
	LQR	0.039	9.5	13.0
	MPC	0.027	8.6	12.5

Dual	PID	0.050	10.8	14.1
	LQR	0.034	8.9	12.3
	MPC	0.023	7.8	11.5

Although LQR and MPC demonstrate lower tracking error and slightly improved energy efficiency, the PID controller offers a compelling balance between control performance and implementation simplicity. In all actuation configurations, PID control was able to maintain acceptable trajectory tracking (RMSE ≤ 0.072 rad) and torque demands within physiological limits, while requiring significantly less computational effort and no predictive modeling, improving compatibility with real‐time embedded applications.

The results show that with careful tuning, PID control provides stable and responsive behavior, even in complex configurations like dual actuation or compliant systems. Notably, elastic and dual actuation systems under PID demonstrated appreciable reductions in peak torque and energy demand, indicating that mechanical design enhancements can complement simpler control strategies effectively.

Given these trade‐offs, PID control was selected as the preferred strategy for this study due to its ease of integration into low‐latency systems, robustness under uncertainty, and feasibility for real‐time deployment in wearable robotic exoskeletons.

### 3.3. Design Verification Using a Virtual Experimentation Model

The virtual experimentation model outlined in Section 2 was employed to calculate the average power consumption of an assistive robotic exoskeleton. A virtual prototype was developed for multiple actuation systems, including rigid, series elastic, parallel elastic, dual rigid, and dual elastic actuation systems.

Since the three variants of the actuation system exhibited similar power consumption patterns, any variant could be used for power assessment. However, the full actuation model with a PWM technique provided a more realistic representation of the system and was thus chosen for evaluating power consumption in rigid, elastic, and dual exoskeleton systems.

#### 3.3.1. Rigid Actuation System

The average power consumption of the rigid actuation system was derived from the virtual model and analyzed in relation to the mathematical model. The analysis covered various transmission system combinations at the lower limb joints.

Figure 10a displays the power consumption results for transmission systems utilizing ball screws and harmonic drives, while Figure 10b depicts the outcomes for harmonic drives integrated with a pulley‐belt drive system, as well as ball screws as transmission mechanisms. This comparison validates the mathematical model and provides insights into power efficiency across different actuation system configurations.

Figure 10Comparison of average total power consumption in a rigid actuation system: (a) utilizing lead screws (B) and harmonic drives (H) as transmission systems, and (b) implementing lead screws (B) with harmonic drives coupled with a belt and pulley system (*H*_B_).

**Figure:**
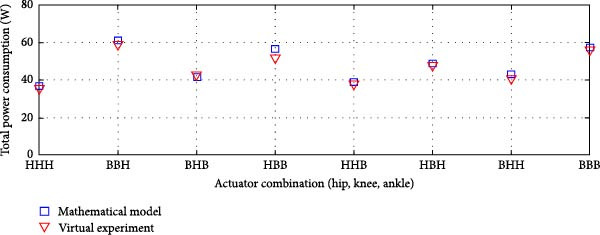
(a)

**Figure:**
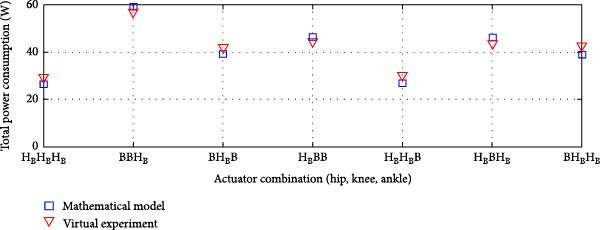
(b)

The total power consumption obtained from the virtual experimentation setup closely matched the values predicted by the mathematical model [25]. A minor variation was observed for the HBB (Harmonic Drive + Belt and Pulley + Ball Screw) combination, but the difference was insignificant.

**Figure 11:**
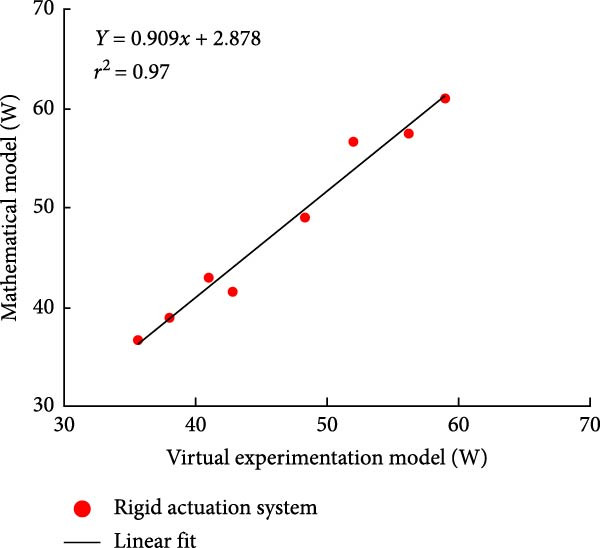
Correlation between average total power consumption in the exoskeleton: mathematical model vs. virtual experimentation for rigid actuation system *r* ^2^: Pearson *R*‐value squared.

In some cases, the virtual model showed slightly higher power consumption than the mathematical model, while in others, it was slightly lower. However, the overall deviation remained minimal. The Pearson *R*‐squared values of 0.97 and 0.96 for the transmission system combinations in Figure 11, respectively, indicate a strong correlation between the two models.

#### 3.3.2. Parallel Elastic Actuation (PEA) System

Figure 12 illustrates the total power consumption obtained from the virtual experimentation setup and the mathematical model for a PEA system. The mathematical model was obtained from our previous study [26]. The power consumption results for the transmission system incorporating harmonic drives and ball screws are illustrated in Figure 12a. Similarly, Figure 13b displays the findings when harmonic drives were integrated with a pulley‐belt drive system or when ball screws were utilized as the transmission mechanism.

Figure 12Comparison of average total power consumption in a parallel elastic actuation system: (a) utilizing lead screws (B) and harmonic drives (*H*) as Transmission Systems, and (b) implementing lead screws (B) with harmonic drives coupled with a belt and pulley system (*H*_B_).

**Figure:**
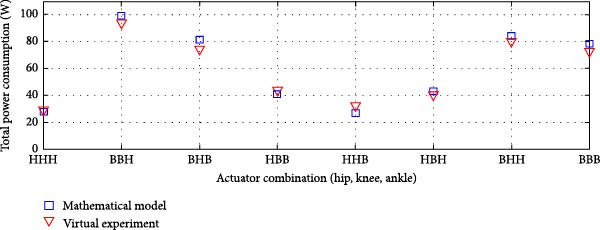
(a)

**Figure:**
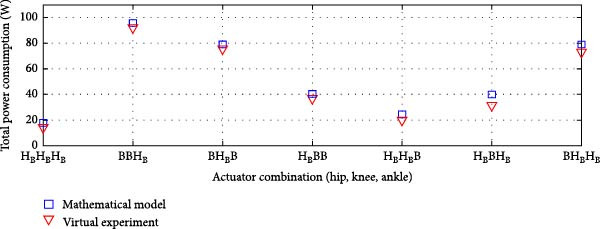
(b)

Figure 13Comparison of average total power consumption in a series elastic actuation system: (a) utilizing lead screws (B) and harmonic drives (*H*) as transmission systems, and (b) implementing lead screws (B) with harmonic drives coupled with a belt and pulley system (*H*_B_).

**Figure:**
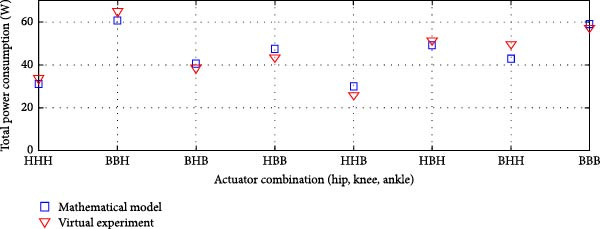
(a)

**Figure:**
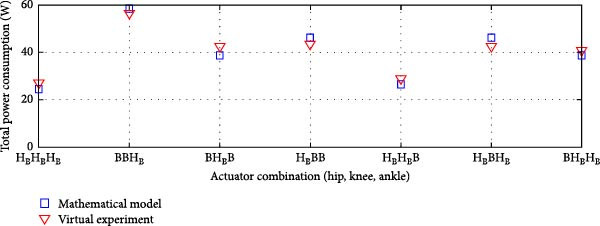
(b)

The PEA system exhibited a power consumption trend similar to that of the rigid actuation system when analyzed against its respective mathematical model. The difference in energy consumption between the two models was minimal. The Pearson *R*‐squared value of 0.99 for both transmission system combinations in the PEA system indicates a strong correlation.

A minor decrease in overall power consumption was noted when harmonic drives were integrated with a belt and pulley mechanism. In an exoskeleton configuration featuring ball screws or lead screws at the hip and ankle joints, along with harmonic drives at the knee, the virtual and mathematical models showed only a slight variation. However, this variation was insignificant, as confirmed by the correlation study. Overall, the total power consumption in the virtual experimentation setup was slightly lower than that predicted by the mathematical model.

#### 3.3.3. Series Elastic Actuation System

Figure 13 displays the average total power consumption of a SEA in an assistive robotic exoskeleton, assessed through a virtual experimentation setup. Figure 13a shows the results for transmission systems using either ball screws or harmonic drives, while Figure 13b depicts the performance when harmonic drives are paired with a pulley‐belt drive system.

The differences between the virtual prototype and the mathematical model were minimal across all transmission system combinations in the SEA. The Pearson *R*‐squared values of 0.89 and 0.95 indicate a strong correlation between the two models. A slight variation was observed when ball screws were utilized at two of the lower limb joints, that is, hip and knee joints, while a harmonic drive was applied to the ankle joint, but the difference was less than 10 W.

When harmonic drives were linked to the belt and pulley system, a lower power consumption was observed, along with a diminished difference between the virtual experiment and the mathematical model. However, the decrease in total power consumption observed in the virtual setup was minimal when compared to the rigid actuation system.

#### 3.3.4. Dual Rigid and Elastic Actuation System

Figures 14–16 present the average total power consumption of a dual actuation system evaluated through a virtual prototype. These figures examine rigid, parallel, and series elastic actuation configurations in a dual setup.

Figure 14Comparison of average total tower consumption in a dual rigid actuation system: (a) utilizing lead screws (B) and harmonic drives (*H*) as transmission systems, and (b) implementing lead screws (B) with harmonic drives coupled with a belt and pulley system (*H*_B_).

**Figure:**
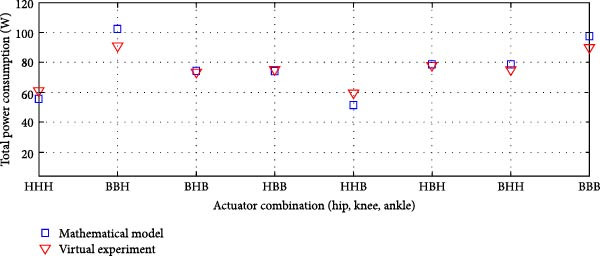
(a)

**Figure:**
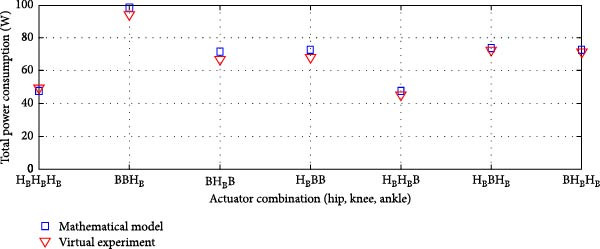
(b)

Figure 15Comparison of average total power consumption in a dual parallel elastic actuation system: (a) utilizing lead screws (B) and harmonic drives (*H*) as transmission systems, and (b) implementing lead screws (B) with harmonic drives coupled with a belt and pulley system (*H*_B_).

**Figure:**
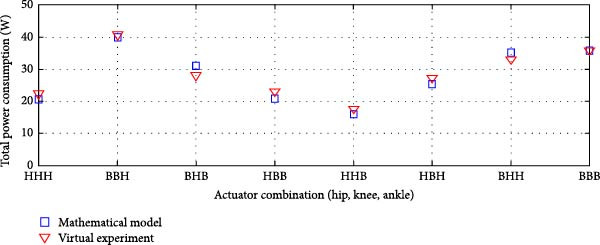
(a)

**Figure:**
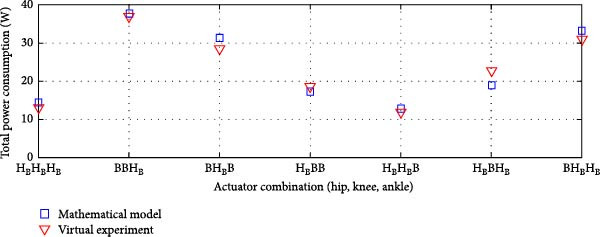
(b)

Figure 16Comparison of average total power consumption in a dual series elastic actuation system: (a) utilizing lead screws (B) and harmonic drives (*H*) as transmission systems, and (b) implementing lead screws (B) with harmonic drives coupled with a belt and pulley system (*H*_B_).

**Figure:**
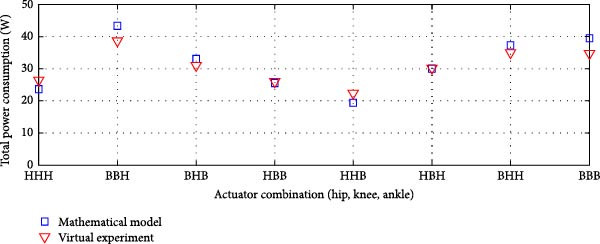
(a)

**Figure:**
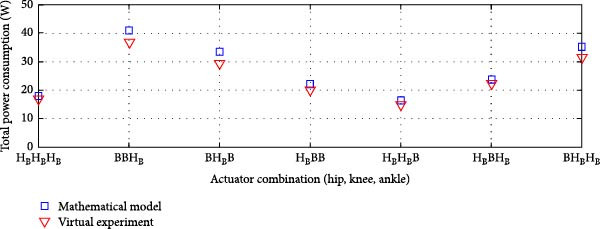
(b)

Figure 14a depicts the power consumption of a dual rigid actuation system employing harmonic drives and ball screws as transmission mechanisms. In contrast, Figure 14b shows the results for systems utilizing ball screws or harmonic drives combined with a pulley‐belt drive transmission. The energy consumption observed in the virtual prototype closely aligns with that predicted by the mathematical model, with only slight discrepancies noted in specific transmission system combinations. Strong correlations are confirmed by Pearson *R*‐squared values of 0.99 and 0.98. Notably, energy consumption decreased when harmonic drives were integrated with a pulley‐belt drive system.

Figure 15 presents the analysis of the average power consumption for a PEA system in a dual configuration. Since one of the primary objectives of the dual arrangement was to adjust spring stiffness during movement, total power consumption was significantly reduced compared to a single arrangement. The results from the virtual prototype closely matched those of the mathematical model, with only slight deviations observed in specific transmission system configurations, such as ball screws at the hip and ankle combined with a harmonic drive at the knee, or a harmonic drive at the hip paired with ball screws at the knee and ankle. The Pearson *R*‐squared values of 0.96 and 0.95 indicate a strong correlation between the two models.

Figure 16 details the findings for a series‐elastic actuation system in a dual setup. The case using ball screws and harmonic drives is illustrated in Figure 16a, while Figure 16b evaluates ball screws and harmonic drives linked with a pulley‐belt drive system. The variable series elastic actuation system did not significantly reduce power consumption, a result consistent with the mathematical model [24]. The Pearson *R*‐squared values of 0.97 and 0.99 indicate a strong correlation between the two models across both transmission system configurations.

Figure 11 illustrates the correlation analysis of the average total power consumption between the virtual experimental setup and the mathematical model for rigid actuation systems. The coefficient of determination (*r* ^2^) demonstrates a strong correlation between the mathematical model and the virtual experimental results, indicating a high degree of linearity. This correlation assessment was conducted across all variations of the actuation systems, confirming the consistency and reliability of the mathematical model in predicting power consumption.

The total power consumption of wearable exoskeleton actuation system was analyzed for rigid, parallel, and series configurations in both single and dual arrangements.

Comparing the optimization results, the most efficient actuator design was identified as a dual actuation system utilizing VPEA with harmonic drives combined with a pulley‐belt drive mechanism at the hip and knee joints and ball screws in an inverted slider crank mechanism at the ankle joint. This configuration minimized energy consumption. However, the addition of an extra motor and transmission system increases the complexity of the system during practical implementation.

While the virtual model in MATLAB Simscape is built upon fundamental physical laws and essentially encapsulates mathematical equations through block‐based representations, comparing it to an explicitly defined mathematical model (e.g., differential equations describing joint dynamics or motor behavior) allows clearer insight into the assumptions, simplifications, and control implications. This comparison helps ensure that the physical fidelity of the virtual model aligns with theoretical control expectations and enables tuning or validating control strategies in both conceptual and practical terms.

### 3.4. Implementation Considerations for Dual Motor Load Sharing

In the dual actuation configuration, two identical motors are employed in parallel to share the mechanical load across each joint. In the simulation environment, load sharing was achieved by supplying both actuators with identical control signals—ensuring synchronized torque generation and halving the effective speed requirement per motor. Translating this strategy into a physical prototype requires precise coordination between the two motors to maintain symmetry and avoid internal stress due to load imbalances.

Real‐world implementation will employ a distributed or centralized embedded control architecture (e.g., dual‐loop PID with feedforward compensation), wherein each actuator is equipped with integrated torque or current sensing. These sensors will enable real‐time feedback to a supervisory controller that actively monitors the load on each motor. Any deviation in torque or speed will be compensated using a differential control scheme, ensuring dynamic load balancing under varying gait conditions. Furthermore, to counter mechanical uncertainties such as backlash or compliance in the transmission, a cross‐coupled control loop may be introduced to enforce coherence between actuators. This ensures robust, synchronized actuation, preserves motor health, and facilitates redundancy, which is advantageous in assistive wearable robotics.

### 3.5. Future Work and Experimental Validation

Although this study was conducted entirely within a virtual prototyping and simulation framework, the presented models and control strategies were developed with real‐world implementation in mind. Future work will focus on the experimental validation of the proposed actuation systems using a physical robotic exoskeleton prototype. The physical system will incorporate DC motors, sensors, and transmission components that correspond directly to those modeled in simulation.

The PID control algorithms used in simulation will be implemented in real time using embedded hardware platforms such as STM32 microcontrollers. Real‐world validation will include joint torque, speed, and power measurements during STS and level‐ground walking tasks. These results will then be directly compared to the simulation outcomes to assess accuracy, reliability, and energy efficiency.

Additionally, although the current models were simulated using nonlinear dynamics in MATLAB Simscape without explicit linearization, future developments will include linearization of the system dynamics around operating points (e.g., mid‐stance during walking or maximum torque during STS). This will enable the application of optimal control strategies such as LQR or MPC, and will help define the operating limits under which these controllers remain valid.

This transition from simulation to real‐world experimentation will serve to validate the energy efficiency and control performance of each actuation mechanism, strengthening the overall impact and applicability of the proposed framework.

### 3.6. Simulation Limitations and Assumptions

While the simulation framework presented in this study allows for detailed analysis of actuation and control strategies, certain simplifications were made. The human user was modeled as a passive load, and no neuromuscular control, user intent modeling, or feedback loops between the exoskeleton and human were implemented. Thus, human–exoskeleton interaction dynamics were not fully captured.

In addition, the environment was modeled as ideal and did not include variability such as ground compliance, disturbances or surface irregularities. Sensors and actuators were also treated as ideal components without accounting for friction, wear, or delay.

These assumptions enabled clearer comparison between actuation mechanisms but do limit the extent to which real‐world behaviors are represented. Future work will expand on these aspects by introducing user‐in‐the‐loop simulations, nonideal components, and environmental dynamics for more robust evaluation and validation.

## 4. Conclusions

This study evaluated the power consumption of an assistive robotic exoskeleton actuation system using a virtual experimentation setup. The performance of different actuation variants, including rigid, parallel elastic, and series elastic systems in both single and dual configurations, was analyzed and compared with results obtained from a mathematical model. The findings revealed a strong correlation between the two approaches, confirming the accuracy and reliability of the mathematical model.

A slight variation in power consumption was observed across specific transmission system configurations; however, these differences were insignificant. The study also highlighted that using harmonic drives combined with a belt and pulley system contributed to a reduction in energy consumption. Furthermore, the dual VPEA system with harmonic drives at the hip and knee and ball screws at the ankle joint emerged as the most energy‐efficient configuration.

Overall, the results validate the mathematical model and support the feasibility of a lightweight, power‐efficient exoskeleton design. However, practical implementation challenges, such as increased system complexity due to additional motors and transmission components, must be carefully considered in future development.

## Disclosure

This study was conducted based on the authors’ interests and utilized computational and experimental resources solely from the authors’ affiliated institutions. All authors reviewed the results and gave their approval for the final manuscript.

## Conflicts of Interest

The authors declare no conflicts of interest.

## Author Contributions

The authors acknowledge their respective roles in this work as follows: study conception and design were carried out by Asim Ghaffar, Muhammad Tanveer Riaz, and Abdur Rehman. Data collection was performed by Asim Ghaffar. Analysis and interpretation of results involved Asim Ghaffar, Muhammad Tanveer Riaz, Abdur Rehman, and M. M. Sayed Al Mahmud. The initial manuscript draft was prepared by Asim Ghaffar.

## Funding

This study did not receive any specific financial support.

## Data Availability

The data that support the findings of this study can be obtained from the corresponding author upon request.
